# The impact of ColRS two-component system and TtgABC efflux pump on phenol tolerance of *Pseudomonas putida *becomes evident only in growing bacteria

**DOI:** 10.1186/1471-2180-10-110

**Published:** 2010-04-14

**Authors:** Marta Putrinš, Heili Ilves, Liisa Lilje, Maia Kivisaar, Rita Hõrak

**Affiliations:** 1 Institute of Molecular and Cell Biology, University of Tartu, 51010 Tartu, Estonia

## Abstract

**Background:**

We have recently found that *Pseudomonas putida *deficient in ColRS two-component system is sensitive to phenol and displays a serious defect on solid glucose medium where subpopulation of bacteria lyses. The latter phenotype is significantly enhanced by the presence of phenol in growth medium. Here, we focused on identification of factors affecting phenol tolerance of the *colR*-deficient *P. putida*.

**Results:**

By using transposon mutagenesis approach we identified a set of phenol-tolerant derivatives of *colR*-deficient strain. Surprisingly, half of independent phenol tolerant clones possessed miniTn5 insertion in the *ttgABC *operon. However, though inactivation of TtgABC efflux pump significantly enhanced phenol tolerance, it did not affect phenol-enhanced autolysis of the *colR *mutant on glucose medium indicating that phenol- and glucose-caused stresses experienced by the *colR*-deficient *P. putida *are not coupled. Inactivation of TtgABC pump significantly increased the phenol tolerance of the wild-type *P. putida *as well. Comparison of phenol tolerance of growing *versus *starving bacteria revealed that both ColRS and TtgABC systems affect phenol tolerance only under growth conditions and not under starvation. Flow cytometry analysis showed that phenol strongly inhibited cell division and to some extent also caused cell membrane permeabilization to propidium iodide. Single cell analysis of populations of the *ttgC- *and *colRttgC-*deficient strains revealed that their membrane permeabilization by phenol resembles that of the wild-type and the *colR *mutant, respectively. However, cell division of *P. putida *with inactivated TtgABC pump seemed to be less sensitive to phenol than that of the parental strain. At the same time, cell division appeared to be more inhibited in the *colR*-mutant strain than in the wild-type *P. putida*.

**Conclusions:**

ColRS signal system and TtgABC efflux pump are involved in the phenol tolerance of *P. putida*. However, as they affect phenol tolerance of growing bacteria only, this indicates that they participate in the regulation of processes which are active during the growth and/or cell division. Single cell analysis data indicated that the cell division step of cell cycle is particularly sensitive to the toxic effect of phenol and its inhibition can be considered as an adaptive response under conditions of phenol stress.

## Background

Aromatic compounds in the environment can either be plant-derived or released as a result of human action. Phenol and other aromatics can be highly toxic, yet their toxicity depends on the concentration of the compound as well as on tolerance level of bacteria. Aromatics such as toluene, xylenes and phenol are harmful, because they dissolve easily in cell membrane, disorganizing its structure and impairing vital functions [1-3]. Disruption of membrane integrity affects crucial membrane functions like acting as a barrier, energy transducer and matrix for enzymes and to certain extent, it also affects cell division and DNA replication. Chaotropic solutes like phenol can also weaken electrostatic interactions in and between biological macromolecules and influence water availability without remarkably affecting cell turgor [4]. When encountering a hazardous aromatic compound, several adaptive responses are triggered in bacteria to neutralize the action of a toxicant. For instance, organic solvent tolerance of *P. putida *relies on several concurrently acting processes: repulsion of solvent molecules, restructuring of cell membrane to reduce harmful effects of the solvent, and active efflux of solvent from the cell [2,5].

Bacterial cell membrane is not only the first target of environmental stress but in many cases it acts also as the first sensor triggering a stress response. The stress signal can emerge from changed membrane properties or from specific signal molecule recognised by a membrane-embedded sensor protein. The ability of bacteria to monitor changes in the environment and to adjust their gene expression accordingly vastly depends on functioning of two-component signal transduction systems (TCS) [6]. TCSs are typically composed of a membrane-located sensor with histidine kinase activity and of a cytoplasmic response protein with a signal-accepting receiver domain. Environmental signal sensed by membrane protein is transduced to a response regulator by phosphorylation.

Bacteria from *Pseudomonas *genus possess tens of different two-component systems. Genes coding for ColRS signal system are conserved in all so far sequenced *Pseudomonas *species http://www.pseudomonas.com indicating its importance in different habitats and environmental conditions. ColRS system was first described in *P. fluorescens *due to its ability to facilitate root colonization by this bacterium [7]. Our studies with *P. putida *have revealed involvement of ColRS TCS in several unrelated phenotypes. First, disruption of ColR response regulator gene resulted in lowered phenol tolerance of *P. putida *[8]. Second, different mutational processes such as point mutations and transposition of Tn*4652 *were repressed in starving *colS- *and *colR*-knockout *P. putida *[8,9]. We associated the latter phenotype with phenol tolerance as the mutation frequency in a *colR*-deficient strain, in contrast to the wild-type, depended on phenol concentration in selective medium [8]. Third, cell population of *colR*-deficient *P. putida *growing on glucose solid medium was heterogeneous: a distinct subpopulation of cells possessed a propidium iodide-permeable cell membrane and a fraction of cells underwent lysis [10]. It is notable that glucose-dependent cell lysis of *colR*-mutant was significantly enhanced if phenol was present in the growth medium [10].

Identification of ColRS-regulated genes has pointed to cell membrane as a potential target of this particular TCS. Namely, the operon locating just downstream of *colRS *genes that codes for a probable lipopolysaccharide kinase and a methyltransferase is positively controlled by ColR both in *P. putida *and *P. fluorescens *[11,12]. In addition, *P. putida *ColRS system negatively regulates transcription of *oprQ *and *algD *genes that code for outer membrane porin and alginate biosynthesis enzyme, respectively [8]. Genome-wide search for ColR regulon in *P. putida *has revealed several other ColR-regulated membrane proteins such as lipid A 3-O-deacylase PagL and diacylglycerol kinase DgkA involved in metabolism of lipopolysaccharides and phospholipides, respectively [12]. Importantly, the presence of phenol in growth medium significantly enhances the effect of ColR on its target promoters [8,12] pointing once more to increased phenol sensitivity of the *colR *mutant *P. putida*.

Many ColR-regulated genes have been tested with respect to their potential participation in the phenol tolerance of *P. putida*. However, despite several efforts we could not identify so far any particular ColR target gene responsible for reduced phenol tolerance of the *colR*-deficient *P. putida *(our unpublished data). Here, to further unravel the role of ColRS system in phenol tolerance, we report on a transposon mutagenesis performed in a *colR*-deficient strain to search for suppressors of phenol sensitivity. This screen disclosed several genes, disruption of which enhanced phenol tolerance of the *colR *mutant. Additionally, we show that phenol sensitivity of the *colR*-deficient bacteria becomes evident only under growth-permitting conditions and not if bacteria are starving for a carbon source. Population analysis at single cell level indicated that particularly cell division is inhibited under condition of phenol stress.

## Methods

### Bacterial strains and media

All strains used in this study are derivatives of *P. putida *PaW85 [13], which is isogenic to fully sequenced KT2440 [14]. To study the role of ColRS system, previously constructed *colR*- and *colS*-knockout derivatives of *P. putida *PaW85, PaWcolR and PaWcolS [9] were exploited. *Escherichia coli *strains DH5α [15] and CC118 λpir [16] were used for DNA cloning procedures, and HB101 [17] as a host for helper plasmid pRK2013 [18]. *E. coli *was grown at 37°C and *P. putida *at 30°C. Bacteria were grown in Luria-Bertani (LB) medium [19] or in M9 minimal medium [20] containing either 10 mM glucose or 10 mM gluconate. Phenol concentrations in minimal media are specified in the text, as they varied between the experiments. When selection was necessary, antibiotics were added at following final concentrations: ampicillin (100 μg ml^-1^), kanamycin (50 μg ml^-1^), or streptomycin (20 μg ml^-1^) for *E. coli *and bezylpenicillin (1500 μg ml^-1^), kanamycin (50 μg ml^-1^), or streptomycin (200 μg ml^-1^) for *P. putida*.

### Selection strategy of phenol tolerant mutants in *colR*-deficient *P. putida *strain

For identification of genes affecting phenol sensitivity, the *colR*-deficient strain was subjected to mutagenesis by Tn5 based mini-transposon containing streptomycin resistance marker. A mini-transposon-carrying plasmid mTn5SSgusA40 [21] was conjugatively transferred from *E. coli *CC118 λpir [16] into a *P. putida colR*-deficient strain with the aid of a helper plasmid pRK2013 [18]. Transconjugants with random chromosomal insertions of the mini-transposon were first selected on glucose minimal plates supplemented with kanamycin and streptomycin. After colonies were grown for three days at 30°C, they were replicated onto glucose minimal plates containing 8 mM phenol. Although a single *colR*-deficient cell could not form a colony on these plates, replication of big and closely located colonies of *colR*-deficient bacteria enabled their growth on replica plates. After another three days, growth of replicated clones in the presence of phenol was evaluated. About 150 transconjugants out of approximately 9000 transposon mutants grew better than *colR*-deficient *P. putida *and they were subjected to secondary assay of phenol tolerance. In order to avoid spontaneous phenol tolerant mutants, the clones of interest were picked up from glucose plates of initial selection. The secondary screen yielded 34 clones with higher phenol tolerance than the parental *colR*-deficient strain. Finally, siblings were eliminated through analysis of clones by arbitrary PCR and sequencing, resulting in 27 independent transposon insertion mutants with elevated phenol tolerance.

### Arbitrary PCR

To identify chromosomal loci interrupted by insertion of mini-transposon in selected clones arbitrary PCR and sequencing were used. PCR products were generated by two rounds of amplifications as described elsewhere [22]. In the first round, a primer specific for the Sm gene (Smsaba - 5'-GAAGTAATCGCAACATCCGC-3') or for the *gusA *gene (Gus2 - 5'-ACTGATCGTTAAAACTGCCTGG) and an arbitrary primer were used (Arb6 - 5'-GGCCACGCGTCGACTAGTACNNNNNNNNNNACGCC-3'). Second-round PCR was performed with primers Smsaba or Gus2 and Arb2 (5'-GGCCACGCGTCGACTAGTAC-3').

### Cloning procedures and construction of bacterial strains

To inactivate the *ttgC *gene in both wild-type and *colR*-deficient backgrounds the *ttgC *gene was first amplified using oligonucleotides ttgCalgus (5'-GAAGAATTCGTCACCCCTGAAAATCC-3') and ttgClopp (5'-CCGAATTCGGTGGGCTTTCTGCTTTT-3') and inserted into EcoRI-opened pUCNotKm (R. Teras). For disruption of the *ttgC *gene in pUC/ttgC, a central 315-bp Eco255I fragment of *ttgC *was replaced with Sm^r ^gene from the pUTmini-Tn5Sm/Sp [23]. The resulting *ttgC*::Sm sequence was cloned from pUC/ttgC::Sm into pGP704L as a EcoRI fragment [24]. Plasmid pGP704L/ttgC::Sm was selected in *E. coli *strain CC118 λ*pir *and the interrupted *ttgC *gene was inserted into the chromosome of *P. putida *PaW85 and PaWcolR by homologous recombination.

For disruption of the *ttgB*, the 5' end of the gene was amplified with oligonucleotides ttgBXba (5'-CAATCTAGAACTGCGCCAGCTCAAGGC) and ttgBSac (5'-CCCGAGCTCTGTTCCATCGAGCGTTTG) and cloned into Eco32I-opened pBluescript KS (Stratagene). The cloned *ttgB *sequence was disrupted by replacing of a central 735-bp EheI fragment with Sm^r ^gene and the resulting *ttgB*::Sm sequence was subcloned into pGP704L as a XbaI-SacI fragment. Finally, the interrupted *ttgB *gene was inserted into the chromosome of *P. putida *PaW85 and PaWcolR by homologous recombination.

### Measurement of unmasked β-galactosidase activity

β-galactosidase activities were measured from solid medium-grown bacteria. As a source of β-galactosidase, the plasmid pKTlacZS/C containing the *tnpA *promoter of the transposon Tn*4652 *in front of the *lacZ *gene, was used [25]. Bacteria grown overnight on solid glucose M9 minimal medium or on the same medium supplemented with 1 mM phenol were scraped off from the plates using plastic swabs. Cells were suspended in M9 solution and optical density of the suspension was determined at OD580. β-galactosidase activity was measured using two alternative procedures. In one procedure, SDS and chloroform were added to the reaction to permeabilize bacterial cell membrane as described previously [26]. In a parallel experiment SDS and chloroform were not added. Percentage of unmasked β-galactosidase activity was calculated by equation: xn/xp × 100%, where xp is β-galactosidase activity measured in assay with SDS and chloroform, and xn is β-galactosidase activity measured using non-permeabilized cells.

### Phenol tolerance assay on solid medium

**Figure 1:**
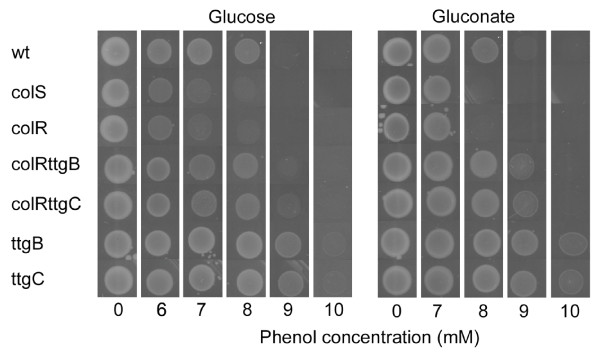
**Plate assay of phenol tolerance**. Results of *P. putida *PaW85 (wt), *colS*-deficient (colS), *colR*-deficient (colR), *ttgB*-deficient (ttgB), *ttgC*-deficient (ttgC), *colRttgB *double mutant (colRttgB) and *colRttgC *double mutant (colRttgC) strains are presented. Approximately 1 × 10^5 ^cells were spotted onto solid medium and plates were incubated at 30°C for 48 hours. The minimal media contained either 10 mM glucose or 10 mM gluconate as the carbon source. Concentration of added phenol is indicated below the figures.

Phenol sensitivity was evaluated on agar plates containing 10 mM glucose or 10 mM gluconate as carbon sources, and up to 10 mM phenol (specified in the Fig. 1). Approximately 1 × 10^5 ^cells were spotted onto plates as 5 μl drops and plates were incubated at 30°C for 48 hours.

### Phenol mediated killing assay

Bacteria were pre-grown on solid glucose minimal plates for 24 hours. Cells were scraped off from the plates and suspended in M9 buffer containing 10 mM glucose and microelements. Optical density of cell suspension was adapted to 0.2 at OD_580_. A number of colony forming units (CFU) was determined directly before and 10, 20 and 30 minutes after addition of 50 mM phenol. Experiment was carried out at 30°C.

### Phenol tolerance microtiter plate assay

Phenol sensitivity was evaluated on microtiter plates containing 100 μl M9 minimal medium in the presence of 10 mM glucose or 10 mM gluconate or in the absence of carbon source. LB-grown overnight cultures were diluted into M9 solution and kept without carbon source for two hours to allow using up any residual carbon and energy source from medium. After that about 5 × 10^5 ^cells per ml were inoculated into the microtiter plates containing different phenol concentrations and appropriate carbon source (if added at all). Microtiter plates were incubated at 30°C with shaking and after 24 hours the CFU was assessed.

### Flow cytometry analysis

*P. putida *cells, grown for 24 h on glucose or gluconate minimal plates with different concentration of phenol, were stained using the LIVE/DEAD BacLight kit (Invitrogen). The kit contains a red fluorescence dye propidium iodide (PI) and green fluorescence dye SYTO9, which both stain nucleic acids. The SYTO9 is able to penetrate all cells, whereas PI enters only the cells with damaged cytoplasmic membranes. If the two dyes are combined then the emission properties of the stain mixture bound to DNA change due to displacement of one stain by the other and quenching by fluorescence resonance energy transfer [27]. Thus, decreased green fluorescence of SYTO9 in the presence of PI indicates entrance of PI into the cells. Staining of cells was performed as suggested by manufacturers and approximately 10 000 events from every sample were analysed with flow cytometer FACSAria (BD Biosciences). Excitation of fluorescent dyes was performed using 488 nm laser. Forward and side scatter (FCS and SSC, respectively) of the light and fluorescence emission at 530 (30) and 616 (26) were acquired for every event. To calculate significance of differences of subpopulations between two strains the Students T-test was performed. Probability was calculated using two-sample equal variance type of T-test and two-tailed distribution.

## Results

### Inactivation of different genes involved in membrane, central metabolism or regulatory functions can increase phenol tolerance of *colR*-deficient strain

The growth of *colR *and *colS *mutant cells is precluded on glucose and gluconate solid medium in the presence of 8 mM phenol, while the growth of the wild-type is not [8] (Fig. 1). However, after few days of incubation of a *colR*-deficient strain on phenol-containing plates, the phenol tolerant mutants appeared with high frequency, approximately 10^-4 ^mutants per cell inoculated (Additional File 1). The high frequency of suppression of phenol sensitivity of *colR *mutant encouraged us to apply transposon mutagenesis for identification of genes implicated in phenol tolerance and potentially interfering in ColRS pathway.

For identification of genes affecting phenol sensitivity, the *colR*-deficient strain was subjected to transposon mutagenesis. Screening of about 9000 transposon insertion derivatives of the *colR *mutant disclosed 27 clones with higher phenol tolerance. Sequencing of mini-transposon insertion sites revealed that phenol sensitivity of the *colR*-deficient strain was elevated by disruption of genes dispersed between different functional classes (Table 1). As ColRS system is obviously involved in membrane functionality [8,11,12] it was expected that disruption of several membrane-related genes could complement the *colR-*deficiency. However, some metabolic genes were also identified as determinants of phenol tolerance (Table 1). Most of mini-transposon insertions were located in open reading frames of targeted genes, thus obviously abolishing their function. However, in case of PP1824 the mini-transposon was inserted upstream of the ATG start codon most probably changing the expression level of this gene.

**Table 1:** Description of chromosomal loci of phenol tolerant mini-transposon derivatives of *colR*-deficient *P. putida *http://www.jcvi.org/.

Locus ID	Gene name	Protein name	Probable localization*	Number of Insertions
PP0145		Na+/Pi cotransporter family protein	CM	1
PP1386	ttgA	multidrug/solvent RND membrane fusion protein	CM	4
PP1385	ttgB	multidrug/solvent RND transporter	CM	9
PP1384	ttgC	multidrug/solvent RND outer membrane protein	OM	1
PP1619		conserved hypothetical protein	C	1
PP1621	pcm	protein-L-isoaspartate O-methyltransferase	C	4
PP1650	gacS	sensor histidine kinase-response regulator	CM	3
PP1842		glutamine amidotransferase, class I	C	1**
PP3997		glycosyl transferase, putative	C	1
PP4422		succinate-semialdehyde dehydrogenase, putative	C	1
PP4798		membrane-bound lytic murein transglycosylase, putative	CM	1

* Abbreviations: CM - cytoplasmic membrane; OM - outer membrane; C - cytoplasm

** insertion 3 bp upstream of ATG

### Disruption of *ttgC *enhances phenol tolerance of both *colR*-deficient and colR-proficient *P. putida*

14 out of 27 phenol tolerant minitransposon derivatives of the *colR*-deficient strain possessed miniTn5 insertion in the *ttgABC *operon (Table 1) and therefore we focused on this system. In toluene tolerant *Pseudomonas putida *DOT-T1E, three homologous efflux pumps TtgABC, TtgDEF and TtgGHI belonging to the RND (resistance-nodulation-cell division) family transporters contribute to solvent tolerance [28]. TtgABC efflux pump plays a major role in antibiotic resistance of this strain, and it also expels solvents and plant antimicrobials from cells [28-31]. The basal expression level of TtgABC in *Pseudomonas putida *DOT-T1E is relatively high being further enhanced by hydrophobic antibiotics and some plant metabolites [30,31]. However, the expression of this efflux system does not respond to solvents [29]. TtgABC efflux pump proteins are highly similar between DOT-T1E and KT2440 strains (over 99% identity) suggesting that their substrate range and biological role could be similar. To find out whether inactivation of *ttgABC *operon only complements the defect of a *colR*-mutant or it can also influence phenol tolerance of the wild-type strain, the *ttgABC *operon was disrupted in the *colR *as well as in the wild-type backgrounds.

In addition to phenol stress, the *colR*-deficient bacteria experience serious glucose-related stress resulting in lysis of a subpopulation of cells [10]. Importantly, cell lysis does not occur on medium with gluconate which is degraded like glucose through Entner-Doudoroff pathway. To test whether inactivation of the TtgABC efflux pump would affect phenol stress only on glucose or it would have a more general role in phenol tolerance, the growth of newly constructed *ttgB- *and *ttgC*-deficient strains were examined both on glucose and gluconate minimal media supplemented with different concentrations of phenol (Fig. 1). In accordance with the transposon mutagenesis screen, the disruption of the *ttgABC *operon made *P. putida colR*-deficient cells more resistant to phenol, and this behaviour was observed on both, glucose and gluconate medium. However, since the *ttgB- *and *ttgC*-deficiency enhanced phenol tolerance also in the wild-type background (Fig. 1), we consider that the TtgABC efflux pump is related to a general tolerance of bacteria to phenol rather than to a particular phenotype of the *colR *mutant.

### Increased phenol tolerance *per *se does not alleviate the phenol-enhanced autolysis of glucose-grown *colR*-deficient cells neither does it restore transposition of Tn*4652 *in the *colR *mutant

**Figure 2:**
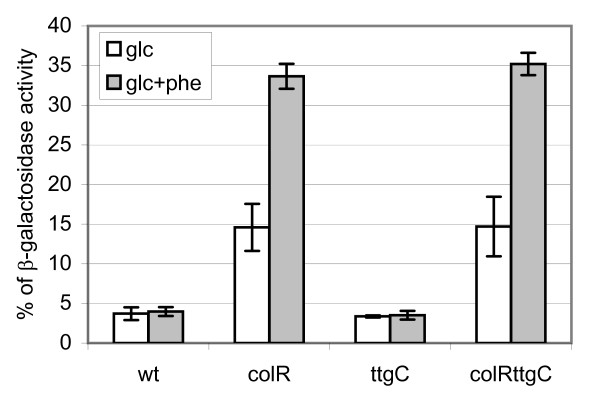
**Unmasked β-galactosidase activity as an indicator of membrane leakiness and cell lysis**. The data present percentage of β-galactosidase activity, measured from non-permeabilized cells against total enzyme activity determined from permeabilized bacteria. Results for *P. putida *PaW85 (wt), *colR*-deficient (colR), *ttgC*-deficient (ttgC) and *colRttgC *double mutant (colRttgC) strains are shown. Bacteria were grown overnight on solid glucose M9 minimal medium (glc) or on the same medium supplemented with 1 mM phenol (glc+phe). Data (mean ± standard deviation) of at least three independent determinations are presented.

In our previous study we showed that phenotypes of the *colR*-deficient bacteria such as membrane leakiness and cell lysis, which are related with growth on glucose, became more prominent if phenol was added to the medium [10]. For instance, glucose-induced release of cytoplasmic β-galactosidase into the growth medium due to the autolysis of the *colR *mutant was significantly enhanced if phenol was supplied [10]. In order to find out whether the increased phenol tolerance can alleviate glucose-induced and phenol-enhanced autolysis of the *colR*-deficient strain, the *ttgC-*knockout derivatives were subjected to β-galactosidase assay. To calculate the percentage of unmasked β-galactosidase activity which was used as an indicator of membrane leakiness and cell lysis, the enzyme activity was measured both in suspension of cells permeabilized with SDS and chloroform (total activity), and in that of intact, non-permeabilized cells. In accordance with our previous results only 4% of total β-galactosidase activity was measurable using non-permeabilized wild-type cells regardless of the presence of phenol in the growth medium [10] (Fig. 2). At the same time, about 15% of total β-galactosidase activity was detectable in case of the *colR*-deficient cells grown on glucose minimal plates, and up to 30% when cells were grown on glucose medium supplemented with 1 mM phenol [10] (Fig. 2). The phenol tolerant *ttgC *single mutant behaved in this test like the wild-type strain (Fig. 2). We expected that enhanced phenol tolerance will reduce the effect of phenol on membrane leakiness and autolysis of the *colR*-deficient strain. However, the *colRttgC *double mutant behaved exactly like its parental *colR *mutant strain in the β-galactosidase assay (Fig. 2). Thus, these data show that increased phenol tolerance of the *colR*-deficient strain acquired by inactivation of TtgABC efflux pump cannot alleviate the effect of phenol as a facilitator of glucose-dependent autolysis.

We have previously shown that transposition of Tn*4652 *is inhibited in starving *colR*-deficient strain when 2.5 mM phenol is used to select transposon insertion mutants that have gained the ability to grow on phenol [9]. Yet, if lower phenol concentrations were used, transposition of Tn*4652 *was somewhat recovered [8]. Therefore, we proposed that increased phenol susceptibility would cause inhibition of transposition of Tn*4652 *in the starving *colR*-deficient bacteria [8]. To test this possibility we analysed the phenol tolerant *ttgC*-knockout derivative of the *colR *mutant in a transposition assay. The transposition assay of the *colRttgC *double mutant showed that despite its high phenol tolerance, transposition was still inhibited like in the *colR *single mutant (data not shown). Therefore, neither the hindrance of transposition nor the glucose-caused cell lysis phenotype of the *colR *mutant correlated with phenol tolerance of cells.

### Survival of the *colR *and *ttgC *mutants in condition of sudden phenol shock resembles that of the wild-type *P. putida*

Our previous study suggested that the *colR*-deficient strain is more sensitive to elevated phenol concentrations due to altered membrane permeability [8]. Propidium iodide staining of glucose-grown bacteria evidenced that a subpopulation of the *colR *mutant possesses indeed highly permeable membrane [10]. In order to clarify whether elevated phenol entrance could cause the lowered phenol tolerance of the *colR *mutant we measured the viability of bacteria that were exposed to high phenol concentration over a short time period. We expected that if phenol entry into the *colR *mutant is increased then the cells of the *colR*-deficient strain should die faster than wild-type cells. Contrary to that, we expected that treatment of the *ttgC *mutant with toxic concentration of phenol will demonstrate long-lasting tolerance of this strain to the toxicant. Unexpectedly, the colony forming ability of bacteria (approximately 10^8 ^cells/ml) exposed to 50 mM phenol did not depend on the functionality of the ColRS system nor the TtgABC efflux pump. After 10 minutes about 70% of the cells were alive independent of their genetic background. By 20 minutes more than 99% of *P. putida *wild-type as well as of *colR*-, *ttgC*- and *colRttgC*-deficient cells were dead (not able to form colonies on selective media) and after 30 minutes of treatment with 50 mM phenol the count of viable cells of all strains had dropped by four orders of magnitude. This data suggests that the cell membrane of the *colR*-deficient strain is not more permeable to phenol than the membrane of the wild-type cells.

### ColRS system and TtgABC efflux pump affect phenol tolerance only in growing bacteria

**Figure 3:**
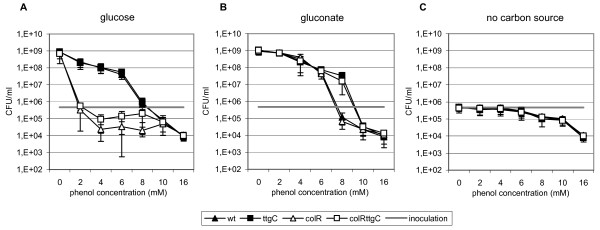
**Liquid medium assay of phenol tolerance**. CFU of *P. putida *wild-type (wt), *colR*-deficient (colR), *ttgC*-deficient (ttgC) and *colRttgC *double mutant (colRttgC) strains in the presence of different phenol concentrations. Phenol sensitivity was evaluated in liquid M9 minimal medium in the presence of 10 mM glucose (A) or 10 mM gluconate (B) or in the absence of carbon source (C). Data (mean ± standard deviation) of at least three independent determinations are presented.

To further investigate variation in phenol sensitivity between the wild-type, *colR*, *ttgC *and *colRttgC *mutant strains we next monitored the 24-hour-viability of bacteria treated with different concentrations of phenol. To evaluate the effect of different physiological conditions, liquid M9 minimal medium contained either 10 mM glucose, 10 mM gluconate or no carbon source at all. As expected, significant differences between the wild-type and *colR*-deficient strains became evident when phenol tolerance was tested on glucose minimal medium. However, differently from solid glucose medium where *colR *mutant is able to grow at phenol concentration as high as 6 mM (Fig. 1), growth of the *colR *mutant in liquid glucose medium was restricted already at 2-6 mM phenol concentration. Moreover, whilst the presence of 4-6 mM phenol allowed growth of the wild-type, then the *colR *mutant started to die at these phenol concentrations and only less than 10% of inoculated cells could survive during the incubation for 24 hours (Fig. 3A). Another interesting phenomenon detected by us was a specific vulnerability of the glucose-grown *colR*-deficient strain to intermediate phenol concentrations (4-8 mM), which is in contrast with its wild-type-like tolerance to high phenol concentrations (10-16 mM) (Fig. 3A). This data correlates well with our finding that the *colR *mutant possesses wild-type-like survival in phenol killing assay (see above) and indicates that in totally stressed cells the phenol tolerance is not influenced by ColRS system any more. Analysis of the *ttgC *mutants revealed that the effect of the *ttgC *disruption on phenol tolerance in the liquid glucose medium was negligible compared to its effect on the solid medium (compare Fig. 1 and 3A). Compared to the wild-type strain, the *ttgC *mutant tolerated higher phenol concentrations on solid glucose medium (Fig. 1) while in liquid medium there were no differences in phenol tolerance between these two strains (Fig. 3A). Also in the *colR*-deficient background the effect of *ttgC *disruption was stronger on solid than in liquid glucose medium (compare Fig. 1 and 3A). Altogether, these results suggest that in the *colR *mutant growing in glucose liquid medium glucose stress prevails over phenol stress.

When phenol tolerance was assayed on gluconate liquid medium, the growth and survival of the wild-type and *colR*-deficient strains did not differ at any tested phenol concentration (Fig. 3B). These results diverge from those obtained on solid medium, where 8 mM phenol enabled growth of the wild-type but not that of the *colR*-mutant (Fig. 1). Thus, in liquid gluconate medium the effect of the *colR *knockout seems to be less pronounced and is possibly detectable only in a narrow window. Comparison of the *ttgC*-proficient and *ttgC-*deficient cells revealed clear differences at 8 mM phenol. While the wild-type and *colR*-deficient strains could not grow at that high phenol concentration and more than 75% of inoculated cells were killed by 24 hours, the *ttgC *mutants survived and even grew at 8 mM phenol (Fig. 3B). Thus, deficiency in *ttgC *increased phenol tolerance of *P. putida *in both liquid and solid gluconate medium.

Surprisingly, in the absence of carbon source, i.e., under growth-restricting conditions, no variations in the viability between the wild-type and the studied mutants were recorded (Fig. 3C). 100% of inoculated cells of all strains were viable in the presence of 4 mM phenol after 24 hours of incubation (Fig. 3C). The number of viable cells of all strains started to drop by increasing phenol concentration, so that only about 2% of cells survived at 16 mM phenol (Fig. 3C). The equal phenol tolerance of non-growing wild-type, *colR *and *ttgC *mutants is in clear contrast with their different behaviour under growth-permitting conditions. However, these results are consistent with our data of survival assay with toxic phenol concentration indicating that permeability of their membranes to phenol is similar. Most interestingly, the *colR *mutant tolerated intermediate phenol concentrations (4-8 mM) in carbon-free medium clearly better than in glucose medium (Fig. 3, compare panels A and C). Thus, presence of glucose remarkably reduces phenol tolerance of *colR*-deficient strain which obviously occurs due to combination of glucose and phenol stress. Contrary to that, availability of glucose as a carbon and energy source significantly facilitates the tolerance of wild-type *P. putida *to toxic effect of phenol, allowing survival of bacteria at 8 mM phenol, i.e., at concentration which kills majority of starving wild-type bacteria (Fig. 3A and 3C). These data are consistent with earlier publications indicating that several energy-demanding mechanisms contribute to fight against phenol by *Pseudomonas *cells [4,32,33]. Hence, our data show that ColRS system and TtgABC pump are involved in phenol tolerance of *P. putida *only under growth conditions indicating that especially growth-related processes of phenol tolerance are affected by both these systems.

### Presence of phenol in growth medium enhances proportion of cells with higher DNA content

Flow cytometry is a technique which allows to analyse microbial population at single cell level and to detect distinct subpopulations with different functional and structural parameters. We have previously shown that population of solid medium-grown *P. putida *is heterogeneous by its DNA content and membrane permeability to propidium iodide (PI) when analysed with flow cytometry [10]. In order to assess how the wild-type *P. putida *and its *colR- *and *ttgC-*deficient derivatives change their population structure as well as membrane permeability when growing on different media supplemented with phenol, the microbial populations were analysed at single cell level.

**Figure 4:**
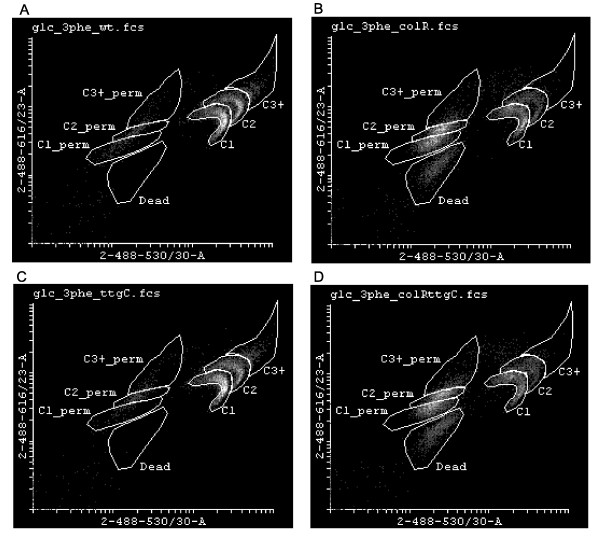
**Visualization of subpopulations by flow cytometry analysis**. *P. putida *wild-type (A), *colR*-deficient (B), *ttgC*-deficient (C) and *colRttgC *double mutant (D) strains, grown for 24 h on glucose minimal plates supplemented with 3 mM phenol were stained using red fluorescence dye propidium iodide (PI) and green fluorescence dye SYTO9, which both stain nucleic acids. Each dot represents an event, analysed by flow cytometer, that has been exicated at 488 nm and respective fluorescence emission has been measured at 530 (30) and 616 (23) nm. Area of seven different subpopulations is indicated. Density plot of results is presented where lighter areas indicated more events with same parameters.

Flow cytometry analysis of solid medium-grown bacteria stained with the mixture of SYTO9 and PI demonstrated highly heterogeneous population structure with seven clearly distinguishable subpopulations (Fig. 4). Cells in the first three subpopulations, named as C1, C2 and C3+, are considered completely healthy as they do not stain with PI. These three populations differ from each other by their SYTO9 fluorescence intensity which most probably reflects their different DNA content. Next three populations, C1_perm, C2_perm and C3+_perm, are considered together as cells with membrane permeable to PI but they can be also distinguished by different DNA content analogous to populations C1, C2 and C3+. This was supported by comparative analysis of SYTO9-only and SYTO9+PI-stained populations which revealed that subpopulations C1, C2 and C3+ observed with SYTO9 alone were equal to the sums of their respective healthy and PI-permeable subpopulations in case of SYTO9 and PI double staining (Additional File 2). Seventh subpopulation, marked as Dead, is clearly present only in glucose-grown *colR*-deficient cells (Fig. 4 and Additional File 3) and correlates with cell lysis. Therefore, this subpopulation most probably represents dead cells with strongly damaged membranes and even lowered DNA content. Latter is supported by observation that glucose-grown *colR*-deficient cells had subpopulation with remarkably lower green fluorescence when stained with SYTO9 only (Additional File 3). In addition, Dead subpopulation has lower side scatter (SSC) indicating that these cells have less complex intracellular structure compared to other cells (Additional File 3).

**Figure 5:**
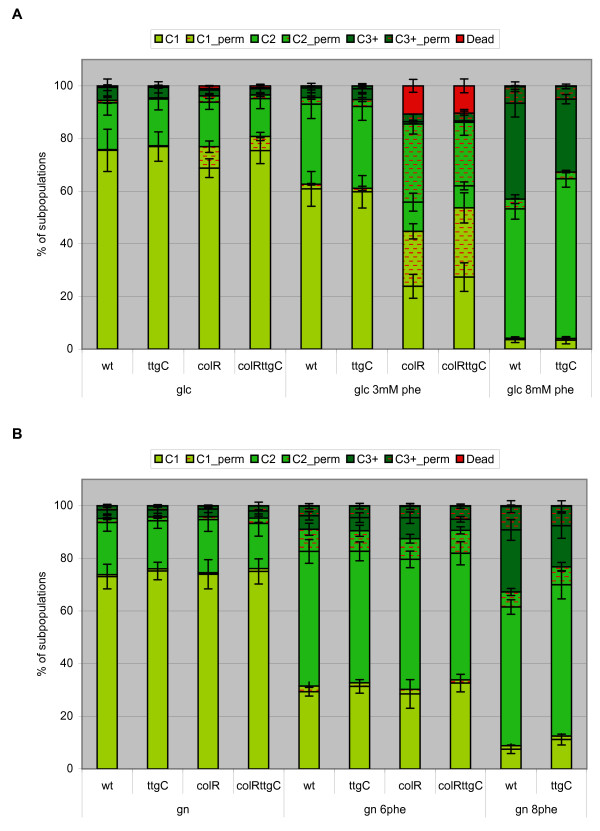
**Cell population structure by flow cytometry analysis**. *P. putida *wild-type (wt), *colR*-deficient (colR), *ttgC*-deficient (ttgC) and *colRttgC *double mutant (colRttgC) strains were grown for 24 h on glucose (A) or gluconate (B) minimal plates. Concentration of phenol (phe) in growth medium (either 3 mM, 6 mM or 8 mM) is indicated below the bars. Cells were stained with PI and SYTO9 and analysed by flow cytometry. Relative proportions of seven subpopulations (as indicated in Figure 4) are shown. Data (mean ± standard deviation) of at least three independent determinations are presented.

Some general observations about the effect of phenol on population structure were made by SYTO9/PI staining and single cell analysis. Most strikingly, independent of *P. putida *strain analysed and carbon source used (glucose or gluconate), addition of phenol to growth medium significantly enhanced proportion of populations C2 and C3+, i.e., those with higher DNA content (Fig. 5), indicating that phenol primarily inhibits cell division and not so much DNA replication. Second, in case of all strains and growth conditions phenol enhanced proportion of PI permeable cells but except for the *colR*-deficient strains grown on glucose this effect was rather modest (Fig. 5). Three PI permeable subpopulations together (C1_perm, C2_perm and C3+_perm) constituted approximately 1-2% of the population of the wild-type and *ttgC*-deficient strain when bacteria were grown on glucose medium. If growth medium was supplemented with 3 mM phenol then the relative amount of PI permeable cells raised up to 5%, and in the presence of 8 mM phenol up to 10% (Fig. 5A). On gluconate the proportion of PI permeable cells was 3-5% in all investigated strains. The presence of 6 mM phenol in gluconate medium increased the relative amount of PI permeable cells up to 15% and 8 mM phenol up to 16% (Fig. 5B). Notably, there were more cells with enhanced membrane permeability to PI among populations C2 and C3+ (containing cells with higher DNA content) than that in C1 population (Fig. 5). As C2 and C3+ cells are those most probably preparing to divide this suggests that temporary enhanced membrane permeability can occur due to cell division.

In accordance with our previous results [10] flow cytometry analysis of the *colR *mutant revealed high amount of cells with membrane permeable to PI when grown on solid glucose medium (Fig. 5A). The presence of phenol in growth medium significantly enhanced proportion of subpopulations with PI-permeable membrane and furthermore, drastically increased subpopulation of dead cells (Fig. 5A). However, these effects are specific to glucose as they do not occur on gluconate medium (Fig. 5B). Thus, the results of flow cytometry analysis confirmed that the *colR *mutant experiences specific membrane leakiness-causing stress only if grown on glucose solid medium and phenol can enhance this phenomenon.

Interestingly, although the wild-type and the *colR *mutant do not differ from each other in respect of proportion of PI-permeable cells when grown on gluconate medium with 6 mM phenol, they still differ if we compare proportions of subpopulations with different DNA content. The phenol-exposed *colR*-deficient strain demonstrates higher amount of cells in C3+ subpopulation than that of the wild-type (Fig. 5B, p = 0.02). From enhancement of C3+ subpopulation with higher DNA content, we concluded, that phenol has stronger cell division-arresting effect on the *colR*-deficient cells than on the wild-type.

Flow cytometry experiments evidenced that the disruption of *ttgC *does not affect cell membrane permeability to PI (Fig. 5). Neither can it affect the proportion of dead cells in the glucose grown *colR*-mutant which is in good accordance with β-galactosidase measurements data (Fig. 2 and Fig. 5A). However, the disruption of *ttgC *affects ratio of subpopulations with different DNA content. On gluconate medium supplemented with 6 mM phenol the amount of cells with higher DNA content (C3+ plus C3+_perm) is lower in the *colR ttgC *double mutant compared to the *colR *single mutant (Fig. 5B, p = 0.027). The effect of *ttgC *becomes evident also in the *colR *proficient background, yet, it occurs at higher phenol concentrations. Compared to the wild-type there are less cells in subpopulations C3+ and C3+_perm of the *ttgC *mutant when cells were grown in the presence of 8 mM phenol on either glucose or gluconate (Fig. 5, p = 0.025 and p = 0.016, respectively). These results suggest that inactivation of TtgABC efflux pump can alleviate the phenol-caused cell division arrest.

## Discussion

Phenol as chaotropic solute can cause different kind of damage such as increase in a leakiness of membrane, enhance oxidative stress, and destabilize macromolecules due to the reduced water activity [4]. Therefore, there are several cellular targets which can be disturbed by phenol. It is known that membrane permeabilizing effect of phenol as well as other aromatic compounds is reduced by rigidification of cell membrane, thus maintaining optimal cell membrane fluidity and permeability [3,34]. Our flow cytometry analysis of phenol-exposed *P. putida *cultures demonstrated that phenol only slightly increased the amount of cells with PI permeable membrane indicating that cells quite well maintain their membrane homeostasis (Fig. 5). Instead, flow cytometry data indicated that the cell division step of the cell cycle is particularly sensitive to the toxic effect of phenol. This was concluded from the finding that the population structure changed essentially if growth medium contained high concentration of phenol. We observed that phenol caused accumulation of cells with higher DNA content indicating cell division arrest (Fig. 5). Phenol is considered to be toxic primarily because it easily dissolves in membrane compartments of cells, so impairing membrane integrity [35]. Considering that cell division and membrane invagination need active synthesis of membrane components, it is understandable that this step is sensitive to membrane-active toxicant, and in this context, inactivation of cell division is highly adaptive for *P. putida *exposed to phenol. In accordance with our findings, literature data also suggest that cell division arrest may act as an adaptive mechanism to gain more time to repair phenol-caused membrane damage. For example, it has been shown by proteomic analysis that sub-lethal concentrations of phenol induce cell division inhibitor protein MinD in *P. putida *[32]. It was also shown that cells of different bacterial species became bigger when grown in the presence of membrane-affecting toxicant [36]. Authors suggested that bigger cell size reduces the relative surface of a cell and consequently reduces the attachable surface for toxic aromatic compound [36]. However, our flow cytometry analysis showed that cell size (estimated by forward scatter) among populations with different DNA content (C1, C2 and C3+) did not change in response to phenol (data not shown). In all growth conditions the average size of cells with higher DNA content was obviously bigger than the size of cells with lower DNA content (data not shown). Therefore, our data indicate that phenol-caused accumulation of bigger cells occurs due to inhibition of cell division which helps to defend the most sensitive step of cell cycle against phenol toxicity.

In this study we disclosed several genetic factors that influence the phenol tolerance of *P. putida*. The finding that disturbance of intact TtgABC efflux machinery enhances phenol tolerance of *P. putida *is surprising because this pump contributes to toluene tolerance in *P. putida *strain DOT-T1E [28,37]. So, our data revealed an opposite effect in case of phenol. In toluene tolerance the effect of TtgABC pump is obvious as it extrudes toluene [28], yet, its negative effect in phenol tolerance is not so easily understandable. Our results excluded the possibility that disruption of TtgABC pump can affect membrane permeability to phenol. Rather, flow cytometry data suggest that functionality of TtgABC pump may somehow affect cell division checkpoint. This is supported by the finding that phenol-exposed population of the *ttgC *mutant contained relatively less cells with higher DNA content than that of the wild-type, implying that in the *ttgC*-deficient strain the cell division is less inhibited by phenol than that in the *ttgC*-proficient strain. Interestingly, the MexAB-OprM pump, the TtgABC ortholog in *P. aeruginosa*, facilitates efflux of a quorum sensing molecule, *N*-(3-oxododecanoyl) homoserine lactone [38]. The TtgABC homologue in *Escherichia coli*, AcrAB-TolC, is also involved in extrusion of quorum sensing signals and in regulation of population entering into stationary phase. Namely, it has been shown that *acrAB-*deficient strain can grow to higher cell density in stationary phase than the wild-type *E. coli *[39] indicating that its cell division is less inhibited by stationary phase factors. In case of *P. putida*, however, we found no evidence that inactivation of TtgABC pump could affect the growth of bacterial culture in stationary phase, as judged by optical density measurements (data not shown). Nevertheless, flow cytometry analysis of the phenol-exposed *P. putida ttgC *mutant revealed population structure indicative of more active cell division than that of the wild-type. However, at this stage of studies we cannot distinguish whether less arrested cell division is a reason for the increased phenol tolerance of the *ttgC *mutant or, *vice versa*, increased phenol tolerance results in less-inhibited cell division.

In our previous study, where we showed that the *colR*-deficient *P. putida *is less tolerant to phenol than its parental strain, we argued that membrane permeability of the *colR *mutant to phenol may be increased [8]. However, results of the current study suggest that the phenol entry into the *colR*-deficient strain is not increased. The latter was supported by the assay which measured the ability of glucose-grown cells to survive in the presence of 50 mM phenol. Unexpectedly, no differences in cell survival between the wild-type and the *colR*-deficient strain were recorded after phenol-shock, indicating similar membrane permeability to phenol in the *colR*-deficient and the wild-type cells. As phenol is known to cause membrane permeabilization [40] we therefore tested whether population of phenol-exposed *colR-*deficient strain could contain more cells with PI permeable membrane. However, as judged by flow cytometry analysis of gluconate-grown bacteria, also the membrane permeabilizing effect of phenol is similar to the wild-type and the *colR *mutant (Fig. 5). Thus, other reasons than enhanced phenol entry or increased membrane permeability should underlie behind the lowered phenol tolerance of the *colR *mutant.

Interestingly, population analysis at single cell level revealed that compared to the wild-type, phenol more efficiently enhanced the relative amount of subpopulations with higher DNA content in case of the *colR *mutant, suggesting that cell division of the *colR *mutant is more sensitive to phenol inhibition than that of the wild-type (Fig. 5). However, it is hard to distinguish whether it occurs due to lowered phenol tolerance or reflects some sort of specific response. Surprisingly, our current study demonstrates that phenol sensitivity of the *colR*-deficient strain drastically depends on whether bacteria are growing or not - no effect of the ColRS system on phenol tolerance of *P. putida *could be detected under conditions of starvation (Fig. 3C). Thus, our data imply that state of metabolic dormancy prevents phenol from hitting its target in the *colR*-deficient cells. We have previously shown that ColR regulates several membrane proteins and is involved in avoidance of several membrane-related disorders [8,10,12]. Therefore it is reasonable to suppose that absence of ColR specifically impairs synthesis or turnover of membrane components and this leads to the reduced phenol tolerance in case of actively growing bacteria. However, in starving cells synthesis reactions are down-regulated and that may cut off the effect of ColR deficiency on phenol tolerance. Such scenario would also explain why differences in survival between the wild-type and the *colR*-deficient strain disappear under growth-permitting conditions at elevated phenol concentrations (Fig. 3A). Eventually, high phenol concentration will totally inhibit biosynthetic processes necessary for cell growth and division, thereby eliminating the target of phenol action in the *colR *mutant.

In addition to increased phenol stress, the *colR *mutant experiences serious glucose-specific stress resulting in cell lysis [10]. Importantly, the presence of phenol strongly enhances glucose-dependent cell lysis of the *colR *mutant as well as proportion of cells with PI-permeable membrane (Fig. 3 and 5). This raises an interesting question about interconnections between phenol- and glucose-caused stresses experienced by the *colR*-deficient *P. putida*. It has been shown by Santos and co-workers that phenol induces expression of proteins involved in cell envelope biosynthesis. Namely, LpxC (UDP-3-O-acyl N-acetylglucosamine deacetylase) and MurA (UDP-N-acetylglucosamine enolpyruvyl transferase) are induced by phenol in a concentration-dependent manner [32]. LpxC and MurA are involved in lipopolysaccharide and peptidoglycane biosynthesis, respectively, suggesting that adaptation to phenol involves higher need for synthesis of cell envelope components. As both pathways use UDP-N-acetylglucosamine, this suggests also enhancement of nucleotide sugar metabolism in response to phenol stress. Considering that lysis of the *colR*-mutant strictly depends on carbon source, the enhancement of glucose-dependent cell lysis by phenol could occur through its dual effect on cell metabolism and membrane homeostasis. Our data suggest that although phenol can significantly enhance the glucose-induced stress in case of the *colR*-deficient strain, nevertheless, the phenol- and glucose-caused stresses are not directly coupled. This was concluded from the cell lysis and membrane permeability measurement data (Fig. 2 and 5) showing that the increased phenol tolerance of the *colR*-deficient strain acquired by the disruption of the *ttgC *gene cannot alleviate the effect of phenol as a facilitator of glucose-dependent autolysis of the *colR *mutant. Our data rather suggest that in the glucose-growing *colR*-deficient strain phenol can activate or inhibit also signals not directly related to its toxicity. It is possible that some kinds of cell growth or division signals are misread in the presence of phenol in the *colR *mutant, which eventually leads to the cell lysis. In that case phenol could act as a signal, leading to the cell death, rather than being killing factor itself. Our further experiments will hopefully clarify whether phenol- and glucose-caused stresses originate from the same defect of the *colR *mutant or they are caused by different reasons.

## Conclusions

Current study demonstrates the involvement of the ColRS two-component system and the TtgABC efflux pump in phenol tolerance of *P. putida*. Our results imply that TtgABC and ColRS systems are not directly connected and may affect phenol tolerance via independent pathways. Both these systems affect phenol tolerance of growing cells only but not of starving ones, indicating that ColRS and TtgABC systems affect processes occurring in metabolically active and dividing bacteria. Most tolerance mechanisms to aromatic hydrocarbons are directed toward maintaining the cell membrane intactness [2]. Given that ColRS and TtgABC systems are also implicated in membrane functions [12,30,38], it is reasonable to conclude that they may assist in regulation of biosynthesis and/or turnover of membrane components, so helping to maintain membrane homeostasis during growth and division. Population structure analysis at single cell level revealed that strong cell division inhibition occurred in phenol-exposed population which could be considered as adaptive response to phenol stress to reduce the phenol-caused damage and to maintain membrane homeostasis.

## Authors' contributions

MP participated in the design of experimental work and manuscript writing. She carried out transposon mutagenesis screen, most phenol tolerance and killing assays, and flow cytometry analysis. HI constructed mutant strains. LL contributed to the mutagenesis screen and phenol tolerance assays. MK participated in manuscript editing. RH performed enzyme measurements and coordinated experimental work and manuscript editing. All authors read and approved the final manuscript.

## Supplementary Material

Additional file 1 **Plate assay of phenol tolerance of *P. putida *PaW85 (wt) and *colR*-deficient (colR) strains**. Cells were grown on glucose (glc) minimal medium in the presence or absence of 8 mM phenol. Approximate number of inoculated bacterial cells is indicated above the figure. Bacteria were photographed after 4 days of growth. Click here for file

Additional file 2 **Comparative analysis of subpopulations with different DNA content by staining of cells with SYTO9 and PI or SYTO9 alone**. *P. putida *wild-type (wt) and *ttgC*-deficient (ttgC) strains were grown for 24 h on gluconate minimal plates supplemented with 8 mM phenol. Cells were stained with PI and SYTO9 (SYTO9+PI) or SYTO9 alone and analysed by flow cytometry. Percentage of subpopulations with different DNA content (C1 and C1_perm, C2 and C2_perm, C3+ and C3+_perm) is shown. Data (mean ± standard deviation) of two independent experiments are presented. Click here for file

Additional file 3 **Description of subpopulation "Dead"**. *P. putida *wild-type (A, C, E) and *colR*-deficient (B, D, F) strains were grown for 24 h on glucose minimal plates supplemented with 3 mM phenol. Cells were stained with SYTO9 alone (A, B) or with SYTO9 and PI (C-F) and analysed by flow cytometry. Fluorescence at 530 (30) is plotted against fluorescence at 616 (23) nm (A-D) or side scatter of light (SSC-A) (E, F). Fluorescence at 530 (30) measures SYTO9 fluorescence and side scatter of light correlates with size of bacterial cells. Click here for file
