# Supplementary figures and images for: The impact of ColRS two-component system and TtgABC efflux pump on phenol tolerance of Pseudomonas putida becomes evident only in growing bacteria
Source: BMC Microbiol. 2010 Apr 14;10:110. doi: 10.1186/1471-2180-10-110 (PMC2865465; doi:10.1186/1471-2180-10-110)

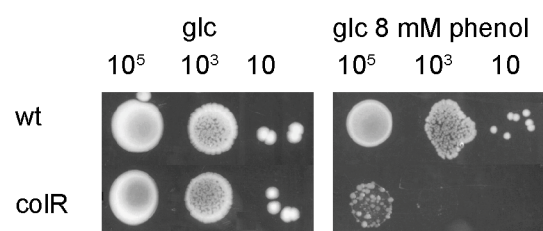

**Supplementary File 1**

Supplement: Additional file 1 — Plate assay of phenol tolerance of P. putida PaW85 (wt) and colR-deficient (colR) strains. Cells were grown on glucose (glc) minimal medium in the presence or absence of 8 mM phenol. Approximate number of inoculated bacterial cells is indicated above the figure. Bacteria were photographed after 4 days of growth. [file 1471-2180-10-110-S1.PDF]

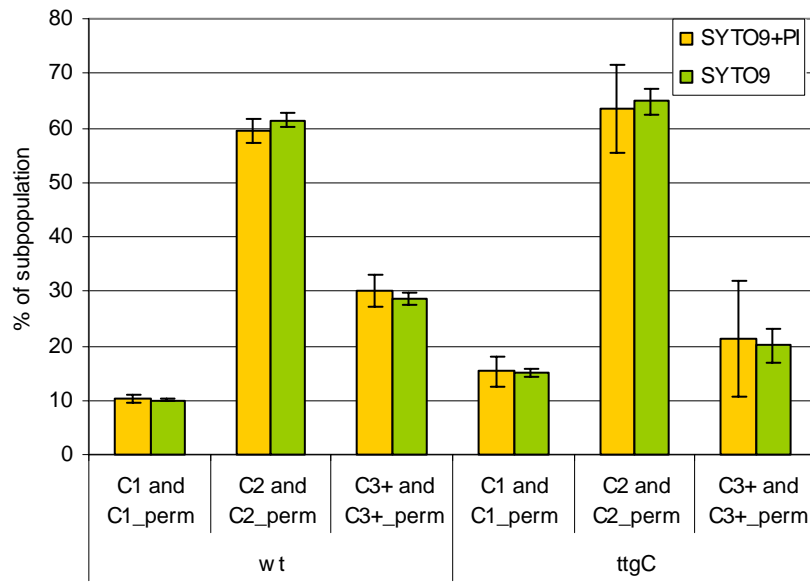

**Additional File 2**

Supplement: Additional file 2 — Comparative analysis of subpopulations with different DNA content by staining of cells with SYTO9 and PI or SYTO9 alone. P. putida wild-type (wt) and ttgC-deficient (ttgC) strains were grown for 24 h on gluconate minimal plates supplemented with 8 mM phenol. Cells were stained with PI and SYTO9 (SYTO9+PI) or SYTO9 alone and analysed by flow cytometry. Percentage of subpopulations with different DNA content (C1 and C1_perm, C2 and C2_perm, C3+ and C3+_perm) is shown. Data (mean ± standard deviation) of two independent experiments are presented. [file 1471-2180-10-110-S2.PDF]

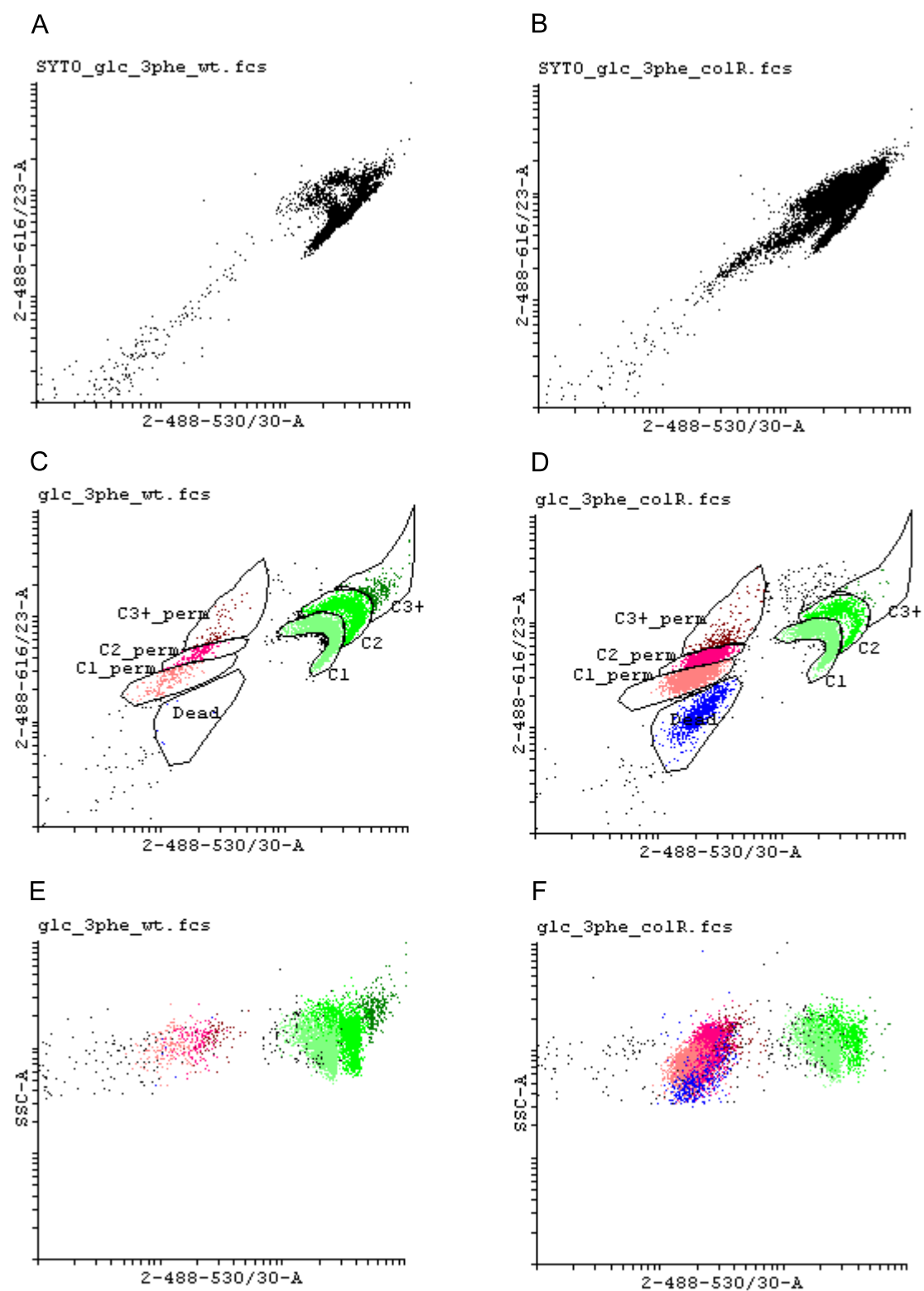

Additional File 3

Supplement: Additional file 3 — Description of subpopulation "Dead". P. putida wild-type (A, C, E) and colR-deficient (B, D, F) strains were grown for 24 h on glucose minimal plates supplemented with 3 mM phenol. Cells were stained with SYTO9 alone (A, B) or with SYTO9 and PI (C-F) and analysed by flow cytometry. Fluorescence at 530 (30) is plotted against fluorescence at 616 (23) nm (A-D) or side scatter of light (SSC-A) (E, F). Fluorescence at 530 (30) measures SYTO9 fluorescence and side scatter of light correlates with size of bacterial cells. [file 1471-2180-10-110-S3.PDF]
